# A Systematic Methodology to Evaluate Prediction Models for Driving Style Classification

**DOI:** 10.3390/s20061692

**Published:** 2020-03-18

**Authors:** Iván Silva, José Eugenio Naranjo

**Affiliations:** 1 Escuela Técnica Superior de Ingeniería de Sistemas Informáticos, Universidad Politécnica de Madrid, 28031 Madrid, Spain; joseeugenio.naranjo@upm.es; 2 Facultad de Ingeniería, Universidad Espíritu Santo, Samborondón 092301, Ecuador

**Keywords:** driving styles, driving styles classification, driving styles methodology, machine learning, intelligent vehicle control, driving safety

## Abstract

Identifying driving styles using classification models with in-vehicle data can provide automated feedback to drivers on their driving behavior, particularly if they are driving safely. Although several classification models have been developed for this purpose, there is no consensus on which classifier performs better at identifying driving styles. Therefore, more research is needed to evaluate classification models by comparing performance metrics. In this paper, a data-driven machine-learning methodology for classifying driving styles is introduced. This methodology is grounded in well-established machine-learning (ML) methods and literature related to driving-styles research. The methodology is illustrated through a study involving data collected from 50 drivers from two different cities in a naturalistic setting. Five features were extracted from the raw data. Fifteen experts were involved in the data labeling to derive the ground truth of the dataset. The dataset fed five different models (Support Vector Machines (SVM), Artificial Neural Networks (ANN), fuzzy logic, k-Nearest Neighbor (kNN), and Random Forests (RF)). These models were evaluated in terms of a set of performance metrics and statistical tests. The experimental results from performance metrics showed that SVM outperformed the other four models, achieving an average accuracy of 0.96, F1-Score of 0.9595, Area Under the Curve (AUC) of 0.9730, and Kappa of 0.9375. In addition, Wilcoxon tests indicated that ANN predicts differently to the other four models. These promising results demonstrate that the proposed methodology may support researchers in making informed decisions about which ML model performs better for driving-styles classification.

## 1. Introduction

Driving styles play an essential role in ensuring road traffic safety and can be defined as the way drivers choose to drive, as well as their associated behaviors and habits developed over the years [1]. Previous literature suggests that an aggressive driving style is one of the most significant causes of traffic accidents [2] that can lead to deaths, severe injuries, and material losses. The World Health Organization (WHO) has reported that 1.2 million people die every year from traffic accidents, and 20 million are severely injured [3]. The American Automobile Association (AAA) Foundation for Traffic Safety [4] has also indicated that an aggressive driving style is the third main concern in road safety in the United States. An aggressive driving style can be defined as the driver’s exhibition of unsafe events, such as speeding, quick lane changes, sudden accelerations and decelerations [5], and a tendency to commit traffic violations [6].

Identifying driving styles may support the provision of effective and customized feedback according to the driver’s performance, offering several benefits, such as reducing energy consumption [7] and ultimately minimizing traffic incidents [8]. Many potential applications that utilize driving-style recognition are being developed [9]. For example, insurance companies are developing automatic ways to ascertain driving styles of their clients in order to offer the right policy [10]. In addition, in the area of advanced driver-assistance systems (ADAS), researchers are interested in the development of intelligent tools to provide tailored feedback according to the driver’s behavior [11].

Current studies have reported different methodologies to classify driving styles using in-vehicle data. These methodologies mostly depend on (i) the inputs that can be extracted and derived from the data gathered (e.g., acceleration, deceleration, brake) [12,13]; (ii) the computational models to classify driving styles [14,15]; (iii) driving-styles outputs (e.g., calm, normal, aggressive) [16,17]; and (iv) the performance metrics that evaluate these models [18,19]. Although these studies show a growing interest in classifying driving styles empirically, few studies have systematically evaluated which inputs and models are better predictors of a particular driving style’s output.

The existing body of research has attempted to evaluate several computational models to identify driving styles using well-established metrics, for example, accuracy, precision, and recall. These studies have reported that Random Forests (RF), Artificial Neural Networks (ANN), k-Nearest Neighbors (kNN), and Support Vector Machine (SVM) are the best classifiers for estimating driver events [14], driving-style maneuvers [19], and driver’s aggressiveness [18]. However, few empirical works have attempted to select the best model across different driving-styles classifiers based on reviewed literature and current research in the field.

A review conducted by Meiring and Myburgh [20] suggested that fuzzy logic and SVM can be considered the most promising simple classifiers (c.f. Occam’s Razor principle [21]) to categorize driving styles. A survey reported by Martinez et al. [9] indicated that fuzzy logic, kNN, and ANN are the most used classifiers to identify driving styles, reviewed from a list of 30 selected articles. However, it remains unclear to researchers which model to choose, as more evidence-based literature is needed in describing how to evaluate these models under the same conditions (e.g., performance metrics, inputs, outputs).

To this end, we propose a six-step methodology, which aims to systematically evaluate different classification models by following best practices in machine learning and artificial intelligence and addressing real-world dataset challenges. In addition, we extend the conventional model evaluation (e.g., classification metrics) of driving-styles classification models by applying statistical tests. To illustrate the methodology proposed, we used in-vehicle data collected from 50 participants, who performed a driving test in a predetermined route in a realistic setting. The in-vehicle data were used to build five models with similar characteristics, as reported in previous works (see Section 3). Human-labeled data from 15 experts was used as ground truth to classify calm, normal, or aggressive driving styles. From the classification metrics, our methodology determined that SVM outperformed kNN, RF, fuzzy logic, and ANN models concerning accuracy (0.96), Area Under the Curve (AUC) (0.973), and Kappa (0.938) measures. Furthermore, results from the Wilcoxon test indicated that the ANN model predicted driving styles in a different way, when comparing the prediction results with the other four models. This work provides an extensive examination of driving styles’ model evaluation, which has been under-explored in this research field.

This article is divided into five sections: Section 2 presents previous works and motivates the need for a systematic approach to allow researchers to get informed decisions about the performance of driving-styles classification models. Next, Section 3 describes the proposed six-step methodology. Section 4 illustrates the proposed methodology by applying each of the six steps to our dataset. Then, Section 5 discusses relevant aspects of the proposed methodology, the experimental results, and limitations. Finally, Section 6 presents the conclusion and sets out promising paths for future work.

## 2. Background and Related Work

In this section, we review related works on driving styles, the concept, external factors that influence it, and the two most common approaches to driving-styles classification. In addition, we describe how previous works have addressed driving-styles classification in terms of data collection, features or inputs, outputs, and type of classification model that has been implemented for this task.

### 2.1. Driving Styles

The field of safe driving has paid considerable attention to the study of driving styles, as this is related to road traffic accidents. As defined by Elander et al. [1], driving styles are referred to as "the way individuals choose to drive or driving habits that have become established over a period of years, including the choice of driving speed, the threshold for overtaking, headway, and propensity to commit traffic violations." The two most common approaches to determine driving styles are self-report and data-driven approaches. Self-report collects driver’s data from questionnaires regarding driver’s motivation or emotions. This is usually understood as the driver state (e.g., stress, anger, inattention, fatigue, drowsiness) [22]. On the other hand, data-driven approaches analyze in-vehicle data in relation to driving events (e.g., braking, turn, lane change) [9]. Data are collected through a myriad of sensors or devices, such as the Global Positioning System (GPS), accelerometers, video cameras, vehicle distance sensors, and on-board diagnostics (OBDII) sensors. In contrast to self-reports, a data-driven approach may be more expensive and harder to collect data en masse, due to the high costs associated with the driver’s data collection (e.g., vehicle, specialized sensors, GPS). However, the results are more accurate because they represent real states of driver’s behavior. In this work, we will focus on a data-driven approach, as we are aware that vehicles are now equipped with modern sensors, and these are becoming cheaper as technology improves.

Sagberg et al. [23] pointed out in a recent review that driver styles are different for each individual, and they represent the stable behavior of a driver during a period. Hence, in this work, we refer to driving styles as any behavior of a driver traveling from point A to point B. Furthermore, driving styles are not just affected by a driver’s behavior, but they also involve external factors, such as traffic flow, road type, time of the day, weather [24], and human factors, such as sociodemographic and general personality factors [25]. For example, the study presented by Ericsson [24] explored how external factors, such as traffic flow and road type, influenced driving styles, as well as time of day (e.g., during day or night). For instance, a driver could exhibit an aggressive style while driving in the city or during peak hours (e.g., managing to drive at high speed); when a road is less busy, the driver could show a calm style (e.g., driving at lower speeds).

As for human factors, significant work has been conducted to associate driving styles (e.g., reckless and careless, anxious, angry, and hostile) with social demographics information, such as gender and education level [25,26,27], using a self-report multidimensional driving style inventory (MDSI) tool. These investigations have shown different driving-style associations across genders and age using the MDSI tool. For example, researchers found that women got stressed (high anxiety levels) while driving, older people drove more calmly (low anger levels) [25], and men drove more aggressively (high reckless and anger levels) [27]. Furthermore, researchers did not find a relation between driving styles and drivers’ socio-economic factors (e.g., education, health, family status). Finally, a recent study revealed a positive association of anger levels with risky driving styles (reckless, anxious, angry) in professional drivers [26].

### 2.2. Driving Styles Classification

Driving-styles classification models are usually built using in-vehicle data to estimate a driver’s style (e.g., calm, aggressive). These classification models can be configured and implemented by considering (1) data collection, (2) feature space or input, (3) output, and (4) a classifier model. This section describes how driving-styles classification models have been implemented in previous work.

#### 2.2.1. Data Collection

Data collection refers to the type of data captured from one or many devices, either from a simulator or a standard vehicle. Simulators can be equipped with a steering wheel, pedals, or a gear shift in order to interact with a virtual environment [16]. Although the use of simulators may provide a better understanding of driving styles in risky situations (e.g., to estimate the driver’s drunkenness or distraction), it becomes an expensive solution, as researchers are required to create a virtual environment that mimics the real world. In contrast, the use of a standard vehicle is becoming more frequent, as different devices can be attached to it for logging the vehicle’s and driver’s data automatically. For example, a GPS device can provide information about the car´s position directly, and speed and acceleration information indirectly [28]. Another popular device is the OBDII, which is connected to a dedicated port in the vehicle and can collect data such as speed, fuel consumption, and throttle [12]. Smartphones are also being frequently used to collect data, as they come with more accessible and reliable sensors, such as GPS, high-definition video-cameras, accelerometers, and gyroscopes [13].

#### 2.2.2. Feature Space or Input

Feature space or input involves feature generation and selection from the devices described above to estimate driving styles. There is no best feature combination to build a model for predicting driving styles, but previous studies revealed that the most common input used for this classification task is acceleration [22,29]. This input is usually combined with other inputs, such as speed [30], deceleration [31], and steering [32]. In addition, longitudinal and lateral acceleration input has been used to capture the way a driver turns or changes a lane [15,33]. Other inputs that are also considered in order to identify driving styles are sudden acceleration and braking [34,35]. In addition, some studies have considered throttle [17], revolutions per minute (RPM) [12], and jerk [36] to estimate aggressiveness. Finally, traffic violations, which are related to traffic accidents, have begun to be considered in recent investigations [13,37].

#### 2.2.3. Output

The output is related to the final categorization or driving-styles classes. Driving styles have been categorized according to aggressiveness levels, with predefined labels, such as calm or aggressive [13,30] and normal or aggressive [17]. Some studies have established an additional label, arguing that drivers may reveal calm and aggressive driving styles at the same time during a route; the driver can, therefore, be labeled as calm, normal, moderate, or aggressive [9,16]. In addition, some studies have reported four or more categories (e.g., a five-point scale from very sporty to very defensive [33] and a four-point scale from below normal to very aggressive [5]), but they are rarely used, as the number of labels impacts the accuracy of the model and affects its interpretation.

#### 2.2.4. Classification Models

Different computational models can be built to classify driving styles. According to a recent review [9,20], the most-used models to identify driving styles are fuzzy logic, RF, kNN, SVM, and ANN. Next, we summarize previous works that have implemented these models.

Fuzzy logic has been used to classify different driving styles (e.g., calm or aggressive [5,10]; comfortable, normal, and sporty [15]) due to its simplicity of implementation and interpretation of results. For example, Castignani et al. [13] classified calm or aggressive driving styles using acceleration, braking, steering, and speeding as features to feed the fuzzy logic system. In their classification model, they used different driver events and external factors, such as weather and time of day, and reported 0.90 model accuracy. In another study, Dörr et al. [13,15] developed an online driving-styles recognition system using simulated data, such as speed, deceleration, acceleration, and time gap, to classify a driver as comfortable, normal, or sporty, with 0.68 accuracy. Aljaafreh et al. [5] designed and tested a model using naturalistic data and the advice of three experts. The model was implemented using three features, namely speed, lateral acceleration, and longitudinal acceleration. These features were used for designing the fifteen rules of the fuzzy logic system, and the output of the system estimates normal or aggressive driving styles. This study did not report any performance metrics of the fuzzy logic model. Although these examples provide a glimpse of fuzzy logic models, there is no consensus on the effectiveness of this model, as these studies have reported different or no classification metrics. In addition, these studies differ in how they gather the data and select the features.

Another strand of studies has implemented ANN models, as well as reporting different accuracy values and configurations (e.g., number of layers and activation function). For example, the ANN model presented in Reference [16] was configured with two hidden layers, with 300 neurons per layer, and the *tanh* activation function. The features that fed the ANN model were acceleration, pedal angle, and speed data gathered from a simulator. This ANN model gave an accuracy of 0.90. Meseguer et al. [12] implemented an ANN with one hidden layer and nine neurons. This model aimed to classify aggressive, normal, or quiet driving styles using speed, acceleration, and RPM data collected from a vehicle. The authors reported an accuracy of 0.77. Jiménez et al. [30] also implemented an ANN model to verify calm or aggressive driving styles based on the vehicle’s energy consumption. The architecture of the ANN was a perceptron network, changing only the weights of the neuron and using speed, acceleration, deceleration, and accelerator pedal data as features. This ANN model achieved an accuracy of 0.83. To sum up, these studies provide a general overview of how ANN models have been implemented for driving-styles classification. None of these studies, however, reported a systematic approach to tune the parameters needed to configure the ANN model.

SVM models have been adopted in this research area due to their suitability to work with real-world problems. Research that implemented SVM models has reported different settings (e.g., kernel function, gamma values), inputs, and accuracy values. For example, Van Ly et al. [31] built an SVM model to identify drivers by modeling driver events (i.e., maneuvers), such as braking, accelerating and turning from acceleration, deceleration, and angular speed features. The model was configured through a Gaussian radial basis function (RBF) kernel, and the authors reported an accuracy of 0.60. Similarly, Zhang et al. [38] implemented an SVM model using a polynomial kernel function and acceleration, throttle position, and engine RPM data as features. This model gave an accuracy of 0.8667. Wang et al. [17] implemented two SVM models to classify normal and aggressive driving styles from speed and throttle opening data. The first model was implemented using an RBF kernel and the second with a linear kernel, reporting accuracy of 0.772 and 0.86, respectively. In short, these studies show several parameter configurations for the SVM model, and the authors reported good accuracy values. Nonetheless, there is a lack of evidence on how to tune the parameters needed to improve the model performance.

RF and kNN models have also been trained and tested for driving-style classification tasks. For instance, Júnior et al. [14] tested different classification models to identify aggressive and non-aggressive driver events from data collected using a smartphone. The authors reported that the RF model (n-trees = 200; m-layers = 10) outperformed the other models by reaching an AUC of 0.99. Similarly, Karginova et al. [39] compared several classifiers, including RF and KNN models, to identify drivers as normal or hard from data collected using an ODB2 sensor. The model that performed better was the RF model (n-trees = 40), giving an overall accuracy of 0.685. Likewise, the kNN model (k = 5) reached an accuracy of 0.68. In another study, conducted by Vaitkus et al. [40], they built a kNN model by analyzing the longitudinal and vertical acceleration data from an inertial measurement unit (IMU) sensor. The authors did not report the number of k, and the model obtained an accuracy of 0.98.

To sum up, several computational models have been implemented for driving-styles classification. However, these studies report different or no accuracy results. In addition, some studies did not report how they selected or tuned the parameters for the model implemented. Hence, it is challenging to know which model achieves better performance for driving-styles classification tasks. To address this challenge, this work aimed to systematically evaluate a set of classification models using well-established classification metrics to assess the output of each model. In order to achieve this aim, we proposed a six-step methodology for driving-styles classification. The next section describes the proposed methodology and its corresponding steps to classify driving styles using in-vehicle data.

## 3. Methodology

**Figure 1:**
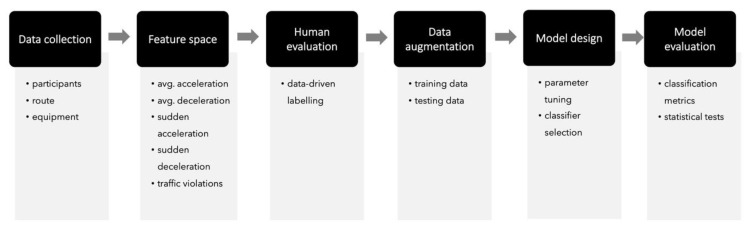
Proposed six-step methodology for driving-style classification.

Motivated by the need for a systematic methodology for driving-styles classification, this work proposes a six-step methodology, as shown in Figure 1. Overall, the methodology is grounded in well-established machine-learning methods and guidelines [41,42] and consists of six steps: (1) data collection; (2) feature space; (3) human evaluation; (4) data augmentation; (5) model design; and (6) model evaluation.

1. Data collection: The first step consists of gathering the in-vehicle data. Depending on the context, drivers are invited to participate in a data-capture session. The driver’s characteristics (e.g., experience, age) will be determined by the aim of the study being conducted. In addition, this step will define the route or routes to conduct driving tests and the equipment needed for capturing and synchronizing in-vehicle and external data. Depending on the research aim, we may consider recording timestamped data (e.g., for time-series or temporal analyses).
2. Feature Space: Based on the data collected during the previous step, several features can be derived. The feature selection is a crucial step for machine-learning models, as these will define the performance of such models [41]. For driving styles-classification tasks, no consensus has been proposed among researchers to convey using a specific set of features. This disagreement is mainly due to the myriad of driving-styles applications (e.g., ADAS development, fuel consumption detection, safety road prevention) and research aims, as these will determine the set of features needed to perform the classification task [9].
3. Human Evaluation: This step involves data evaluation by experts to get the ground truth. This step is a standard procedure to follow, when the overall purpose is to mimic experts’ decisions through automated algorithms or models. Usually, two or more raters (experts) inspect the data and assign a category or a score to each instance (a driver, in our particular case). The final category or score from each rater is then processed to calculate an inter-rater agreement score (e.g., inter-rater reliability (IRR) coefficient). A low score means that experts did not get similar results in the dataset evaluation, whereas a high score means that experts achieved a similar evaluation. Experts should iterate over their evaluation until they reach a good inter-rater agreement score.
4. Data augmentation: A common classification problem in real-world scenarios is the lack of big-data availability, forcing researchers to work with small datasets, which in turn can be noisy or present unbalanced class distribution. A way to address this challenge is to produce synthetic data through a generative model, based on the original dataset. This process is called data augmentation [43] and takes the human labeled data to generate new datapoints that are close to the real data points. The data-augmentation process results in a new dataset, which can be used for model training purposes.
5. Model design: Most machine-learning models need to be *designed* in order to find the best prediction model for the particular problem. Researchers should be aware of the configuration needed to implement a model. Some configuration steps require expert knowledge, for example, the design of fuzzy logic membership functions. Other configuration values can be defined semi-automatically by performing a grid search on model parameters. For example, in SVM models, the kernel function, C, and gamma parameters are crucial to find a model that performs a good classification task. To this end, prior research has developed a set of guidelines to implement SVM models and define such parameter values [44], which includes a grid search process.
6. Model Evaluation: As suggested by Sokolova and Lapalme [45], several classification metrics can be derived from the confusion matrix in order to evaluate and compare the performance of several machine-learning models. These classification metrics correspond to accuracy, specificity, recall, F1-score, the Area Under the Curve (AUC), Kappa, and so on. Accuracy is the most used metric in machine-learning problems to determine the precision of a model according to its correctly classified examples (from now this relates to each driver) and the total size of the dataset. Another useful metric that considers the dataset class distribution (e.g., balanced vs. unbalanced data) is the F1-score. This metric is helpful as it takes false positives and false negatives to determine the performance of a model, which is essential in real-world datasets. In addition, the AUC is used to determine whether a model is capable of differentiating among classes by comparing the rates of false-positive and true-positive instances. As opposed to the accuracy, this measure does not consider dataset size to evaluate the performance of the model. Finally, the Kappa statistic measure considers the observed and expected accuracy in evaluating the performance of the model, which is more robust than merely relying on accuracy. This Kappa results in a measure that evaluates the agreement between model output and the ground truth.

Another way of comparing the performance of the classification models is employing a statistical significance test. The purpose of these tests is to know in which proportion the results are representative of the general behavior of several classifiers [46]. Paired tests are commonly used to compare whether each pair of classifiers predicted in a similar or equal way, meaning that both classifiers came from the same solution space.

## 4. Illustrative Study

We conducted a study to illustrate each of the six steps proposed in our methodology. These are explained in the sections below.

### 4.1. Data Collection

In this section, we detail the information related to the data-collection step in a realistic setting, including participants’ information, the selected routes, and the equipment used to collect data during driving tests.

#### 4.1.1. Participants

A total of 50 drivers (31 males, 19 females, avg. 48.24 years, SD 10.61) participated in a driving test. Drivers had an average driving experience of 15.78 years (SD 10.01). In terms of age, 22 drivers were between 25 and 34 years, 19 drivers between 35 and 54 years, and 9 drivers between 55 and 65 years. All drivers had a non-professional driver’s license and performed the driver test in a small or medium vehicle. Before starting driving tests, participants were asked to drive in the small or medium vehicle as they usually do for a short period to get used to that vehicle (e.g., change velocity). A researcher was present as co-pilot during driving tests to give instructions to drivers about the route to follow.

#### 4.1.2. Route

We selected two similar routes, one in Guayaquil (40 driving tests) and another in Madrid (10 driving tests). Both routes had a duration of approximately 24 min and an average distance of 18 kilometers. In addition, both routes had 23 speed-limit signs (e.g., 40 km, 70 km, 90 km), and included different types of route (e.g., highways, suburban roads, and urban roads). All the tests were done during the day with different traffic flow.

#### 4.1.3. Equipment and Data

Data from traffic signs were logged for the experiment. One of the researchers drove the selected route and logged in the GPS position of the traffic signs with a smartphone and the traffic sign value (e.g., 50 km/h). As this step was manually done, we foresee that automatic data would be captured using additional sensors (e.g., extract traffic signs from real-time video cameras, accessing a web service) in the near future.

Two devices were used to collect in-vehicle data: an OBDII device, plugged in to the car, and a smartphone with the Torque application installed (Available at https://play.google.com/—Torque Pro) to receive OBDII data via Bluetooth. The OBDII captured and sent the vehicle’s speed data at a sampling rate of 1 Hz. The Torque application saved the smartphone GPS position (latitude and longitude) data and the timestamp for each record. Hence, we assume that the smartphone position is the vehicle position.

### 4.2. Feature Space

The instant acceleration (m/s^2^) was calculated from the vehicle’s speed data (km/h). Then, four features were derived from instant acceleration.

The first two features were the average acceleration (AvgAcc) and deceleration (AvgDec) from the whole route. The third and fourth features corresponded to the total occurrences of sudden acceleration (EventAcc) and sudden braking (EventBrake) events during the route. These two features were calculated according to a set of threshold values for instant acceleration, as reported in Reference [47]. We considered that drivers do not exhibit the same behavior in a congested route as in a free route. Therefore, we established three traffic-flow levels to derive these features: low, medium, and high. As shown in Table 1, different threshold values were selected for the three traffic-flow levels. Thus, in order to count the total number of EventAcc or EventBrake, first, we set the traffic level of the route (i.e., low, medium, high), which was defined by the researcher that was present during driving tests. Then, we counted how many times the instant acceleration had a value equal to or greater than its corresponding threshold for EventAcc and EventBrake.

For example, if driver A performed driving tests in an overcrowded route, the researcher assigned a high traffic-flow level. With the data collected from driver A, we calculated the instant acceleration and counted the times this value was equal to or higher than its corresponding threshold of 1 m/s^2^. This value corresponded to the total occurrences of sudden acceleration events. Similarly, we counted the times the instant acceleration was equal to or lower than its threshold of −1 m/s^2^. This value corresponded to the total occurrences of sudden braking events.

Finally, a fifth feature, traffic violations, was derived from the data collected from each driver. To obtain traffic violations, we modeled a fuzzy logic system to estimate traffic violations from in-vehicle data, as reported in a previous work [37]. The model takes two inputs: (1) the driver’s speed excess, which is calculated from the difference between the speed limit and the speed of the vehicle when it is near to a traffic sign; and (2) the severity of the traffic violation, which is determined by experts and speed-limit laws. The output of the model is a value between 0 and 1, where a value of 0 means that a driver had a moderate level of traffic violations, and a value of 1 means that a driver had a high level of traffic violations. For further details of the model, see Reference [37].

### 4.3. Human Evaluation

Human experts were asked to evaluate each driving test by categorizing each driver as calm, normal, or aggressive to determine driving styles. Then, 15 external experts, with a broad experience in automobile research, categorized each driver following a data-driven approach (e.g., by analyzing the feature space). The inter-rater reliability (IRR) coefficient from the 15 evaluations yielded a value of 0.958. This coefficient suggests excellent reliability for the evaluation made by humans [48]. We took the median value as the final class for each driver. The class distribution ended up with 10 calm, 22 normal, and 18 aggressive drivers.

### 4.4. Data Augmentation

The Synthetic Minority Over-Sampling Technique (SMOTE) was applied to our dataset. It aims to generate synthetic data and preserve the original distribution. This is done through the creation of a new data point through perturbing an instance [49]. Using Weka (Available at https://www.cs.waikato.ac.nz/ml/weka/downloading.html) [50], we applied the SMOTE filter to our dataset. The new dataset comprised 165 new instances (55 per class). It is worth noting that for this classification problem, the augmented dataset will be used as a training set, whereas the original dataset will be used as a test set.

### 4.5. Model Design

In this section, we explain how we designed and built the five models, regarding parameter tuning and other inherent characteristics for each model. We selected five types of models based on prior literature and relevant works (Section 2.2.4), which indicate that fuzzy logic, ANN, SVM, kNN, and RF are the most common models being used for driving styles classification. Each of these types of model was trained and tested using combinations of parameters, creating a subset of models. From this subset, the model with the best performance (i.e., in terms of accuracy) was selected. Therefore, one model was selected per type of model. It is worth noting that the design and parameter tuning of the models was performed using the data generated from the previous step, which is the augmented dataset.

#### 4.5.1. Fuzzy Logic

The design of the fuzzy-logic model followed a data-driven approach, motivated by previous works (see Section 2) and the advice of experts. Our fuzzy model was designed using a Sugeno FIS, and it was implemented using the MATLAB tool (Available at https://www.mathworks.com/products/matlab.html). We decided to use Sugeno FIS because it gives faster results than other FIS [51,52]. Fuzzy-logic models work with linguistic labels to represent the fuzzy values of the problem domain. In this case, the linguistic labels corresponded to low, normal, and high for all the five inputs. In addition to this, we defined: (1) the membership functions of the five inputs; and (2) a set of rules to represent the fuzzy model based on the inputs and the linguistic labels.

**Figure 2:**
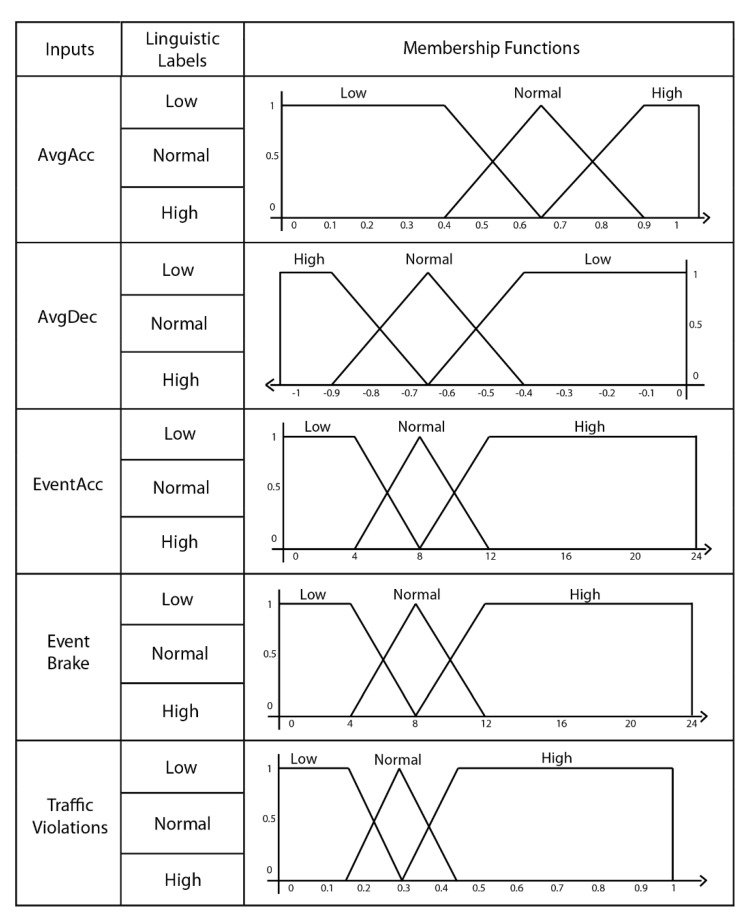
Input variables description with their linguistic labels and membership functions.

Membership Functions: We applied triangular and trapezoidal membership functions due to the easiness of assigning interval values to linguistic labels. Figure 2 depicts the membership functions for each of the five inputs. As can be observed from Figure 2, the AvgAcc input is composed of three membership functions, each one corresponding to a linguistic label: a trapezoidal function for low with a range of values from 0 to 0.65; a triangular function for normal with a range of values from 0.4 to 0.9; and a trapezoidal function for high with a range of values from 0.65 to 1.2. The other inputs were similarly composed.

Output: For the output of driving styles, we determined three linguistic labels with their constant membership function. The linguistic label calm has a value of 0 for the constant membership function, the normal label has a value of 0.5, and the aggressive label has a value of 1. As the fuzzy model gave values between 0 and 1, we assigned ranges to determine the final class. For instance, if the estimated value was between 0 and 0.33, driving styles were classified as calm. Alternatively, if the estimated value was between 0.34 and 0.66, driving styles were classified as normal. Finally, if the estimated value was between 0.67 and 1, driving styles were classified as aggressive.

Rules: The simplicity and easiness of a fuzzy-logic model are given by the number of rules. For this problem, we defined 10 rules to classify the driving style (Table 2). Each rule is composed of an antecedent (the input and its assigned linguistic label), a consequent (the model output), and a weight. The weight determines the relevance of the rule, which, in this case, is critical to identify aggressive drivers, as they may need more support for changing their behavior. Thus, we assign a weight of 0.8 when the consequent was calm, a weight of 0.9 when the consequent was normal, and a weight of 1 when the consequent was aggressive. After training and testing the fuzzy-logic model, this model yielded an accuracy value of 0.88.

#### 4.5.2. Artificial Neural Networks

Two factors should be considered when designing ANN models: (1) the selection of the training algorithm; and (2) the network architecture [53]. In this work, we selected the backpropagation algorithm as the training algorithm, because it had shown good classification results [12,33]. For the network architecture, the following parameters should be tuned: (i) the number of hidden layers; (ii) the activation function; (iii) the number of max iterations; (iv) the learning rate (*lr*); and (v) the number of neurons (N) for the hidden layer.

We implemented an ANN model using the RStudio tool and the RSNNS library [54]. Following a similar design, as reported by Hornik et al. [55], we implemented an ANN model with one hidden layer and the sigmoid activation function. In order to find the model with the best performance, we trained and tested several ANN models with different parameter values. Thus, we performed a grid search to tune the number of max iterations, the learning rate, and the number of neurons. We set the max iterations to 400, as we observed that the error converged with this value. The learning rate was selected from a range between 0.1 and 1, and the number of neurons was selected from a list of values (3, 4, 10, and 12) as suggested in several studies [56,57,58,59] (Table 3). The best combination of parameters that produced the highest accuracy (0.86) was a learning rate value of 0.4 and four hidden neurons.

#### 4.5.3. Support Vector Machines

Support Vector Machine is a supervised learning model that was introduced in 1992 by Boser et al. [60]. It aims to find the optimal hyperplane that separates two classes (e.g., positives vs. negatives) by finding the maximum margin distance. This separation can be done in the same feature space, or in a high-dimensional space through the mapping of the input space into a feature space by using a kernel function data (e.g., RBF, polynomial) [61]. In addition to this, SVM adjusts the trade-off between the margin and fitting error by modifying a C parameter. Following the guidelines provided in Reference [44,62], we performed a grid search to tune the model by choosing (1) the kernel function, and (2) the cost-function (C) and γ hyper-parameters. Thus, we tested every possible combination of parameter values and kernel functions. The SVM models were implemented using the RStudio tool and the LIBSVM library [63]. Table 4 lists the four kernel functions and the range of values for the C and γ parameters that were used to tune the parameters. The accuracy was considered to evaluate the performance of the model. An SVM model configured with an RBF kernel yielded the highest accuracy (0.96). As several combinations of C and γ values performed equally (i.e., the same accuracy), we decided to choose the lowest value for C (2^5^) to avoid over-fitting and an intermediate value for γ (2^2^) [64].

#### 4.5.4. Random Forests

Random Forests (RF) are an ensemble learning method where several decision trees are trained and the final class prediction is the mode or average of the decision trees. The intuition behind RF models is that a large number of uncorrelated models outperforms any individual decision tree model. In order to ensure uncorrelated decision tree models, RF applies bagging and feature random selection [65]. In bagging, the decision trees are trained using a random sample size with replacement. Therefore, this method results in different decision trees with a lower variance of the model. As for random selection, the decision tree uses a random subset of features, resulting in more diversification. Two parameters should be tuned to implement an RF model: (1) the number of trees (n) to grow in the forest and (2) the number of layers (m) to split the nodes of each tree. The RF model was implemented using the RStudio tool and the randomForest library [66]. As suggested by Breiman [67], a large number of n makes the model converge to an error of zero. Moreover, choosing a larger value of m increases the correlation between trees producing similar results in the voting. We performed a grid search to select the best values for n and m, using values between 1 and 500, and between 1 and 10, respectively. The RF model configured with an n = 100 and m = 4 yielded the highest accuracy (0.92).

#### 4.5.5. kNN

A non-parametric method used for classification and regression, which has been considered the simplest of all supervised classification models. This algorithm relies on the idea of similarity, meaning that observations (points) with similar characteristics generate similar results [68]. The similarity between two observations is calculated using a distance, proximity or closeness function. Thus, an observation is classified by assigning the most common class among its k nearest neighbors. To implement the kNN model, we needed to configure (1) the number of neighbors (k), and (2) the distance function, in which the most common is the Euclidean function [69]. Choosing the k is not an easy task, as it may affect the accuracy of the classification results. Therefore, a grid search was performed to find the best k for this dataset using values between 1 and 10. A value of k = 3 produced the best performance, with an accuracy of 0.92.

### 4.6. Model Evaluation

Once we had selected the models with the best performance for each classifier, as described in the previous section, we evaluated the performance in terms of (a) classification metrics (e.g., accuracy, recall, Kappa); and (b) statistical test (e.g., Wilcoxon test), which is summarized in the following section.

#### 4.6.1. Classification Metrics

We calculated the confusion matrix for each model. According to well-defined guidelines [45,70], selecting the metrics to evaluate the performance of our models depends upon the dataset and its characteristics (e.g., balanced vs. unbalanced data, type of data). As we are dealing with an unbalanced data set, the accuracy is not enough to evaluate the performance of the five models. Thus, accuracy, F1-score, AUC, and Kappa measures were selected to compare and determine the best model that suits this dataset. Table 5 presents the results of accuracy, F1-score, AUC, and Kappa for each model. We stress in bold the highest values for each metric. According to these results, the SVM model with a RBF kernel (C = 2^5^; γ = 2^2^) outperformed all other models in relation to all metrics.

Although these results are promising regarding the overall performance of the five models, in this work, aggressiveness had a higher impact in a real-world setting, as an aggressive driver is prone to cause more traffic violations and accidents than a normal or calm driver. Therefore, we were interested in exploring the classification performance for each class, with a particular emphasis on the aggressive class. Table 6 compares the F1-score and AUC per class. As can be observed from this table, SVM, RF, and kNN models have the same performance for calm driving styles (F1 = 0.9524; AUC = 0.9875). However, SVM outperformed the other models regarding normal (F1 = 0.9545; AUC = 0.9594) and aggressive (F1 = 0.9714; AUC = 0.9722) driving styles.

#### 4.6.2. Statistical Tests

In this research, we used the Wilcoxon signed-rank test, which assumes non-normality in the data distribution and it is utilized to compare the differences between two samples that are paired or related [71] Wilcoxon test was applied with all possible combinations among the five models considered in this work. For this test, the null hypothesis (Ho) states that there is a significant difference in how the model predicts (*p* ≤ 0.05). The p-values resulting from the paired test, as shown in Table 7, indicate that ANN predictions are significant regarding the other four models (*p* = 0.025 for all ANN paired tests.

Relevant findings from these results, as well as implications of the proposed methodology, are discussed in the next section.

## 5. Discussion 

Analyzing driving styles through self-reports can be inaccurate as only the driver’s perspective is considered to determine the driver’s aggressiveness [72]. Researchers in this area have proposed computational approaches to detect driving styles from in-vehicle data automatically. This work presents a data-driven approach to classify driving styles as calm, normal, or aggressive by training and testing five models, following a systematic methodology, and finding the best model using machine-learning performance metrics and statistical tests. Here, we discuss our results in terms of (1) the six-step methodology proposed for driving-styles classification; and (2) the results obtained from the evaluation, once we applied the proposed methodology to our naturalistic dataset.

### 5.1. A Six-Step Methodology for Driving-Styles Classification 

The methodology proposed in this work was motivated by well-established methodologies and guidelines for machine-learning approaches. In order to get a good driving-style classification model, a compelling methodology is needed to validate the experimental results. From previous works discussed in Section 2.2.4, we noticed that most of the driving-styles classification models reported lacked thorough validation. Therefore, we expect that this methodology will contribute to the current state of the art by guiding researchers in implementing a standardized way of selecting and evaluating classification models.

We recognized some aspects that were relevant in the proposed six-step methodology to build and select the model with the best performance for driving styles classification, as discussed below.

In terms of data collection, the proposed methodology was illustrated using data from 50 drivers performing a driving test in a naturalistic setting. In-vehicle data from two different contexts with similar characteristics were analyzed. Previous studies have reported the implementation of classification models using data gathered from simulators [15,32]. Although this could be an advantage for predicting behaviors in dangerous driving situations, it is preferable to use data captured from real scenarios. In this work, we demonstrated that data from a realistic setting could be correctly classified as calm, normal, or aggressive driving styles with an accuracy of 0.96 with the SVM model.

In terms of data labeling or ground truth, similar studies for driving-style classification have considered self-report measures through questionnaires (e.g., the Driving Behavior Questionnaire—DBQ) or tickets from traffic violations as a way of categorizing drivers and their driving styles [35]. These approaches may not be considered the best solution, given that they rely on subjective data [72]. On the other hand, prior studies have come up with more straightforward methods to get the ground truth of in-vehicle data. For instance, Jiménez et al. reported the creation of their own dataset by asking drivers to change their driving style into calm or aggressive before data collection [30]. Dörr et al. utilized three simulated virtual scenarios to label their dataset as comfortable, normal, or sporty driving styles [15]. Another set of studies reported the use of unsupervised machine-learning models, such as K-means, to find common group patterns that can be related to driving styles, such as calm or aggressive [13] and normal or aggressive [17,18].

By contrast, our study provides a common practice in machine-learning research by labeling that data manually with the help of experts in the field. This approach is known in transportation research as a data-driven approach and its validation relies on the number of experts involved and the inter-rater reliability reached among experts. The data-driven approach has not been widely explored for driving-styles classification. This can be due to the time-consuming task of labeling the data case by case and the costs associated with this task. For instance, we only found one study where experts applied a data-driven approach to detect driving styles, such as sporty, normal, and defensive. However, the authors did not report how many experts were involved in the data labeling and the inter-rater reliability reached among experts [33]. In our study, 15 experts labeled the dataset, by assigning a class (i.e., calm, normal or aggressive) to each driver. As indicated in Section 4.3, we obtained higher inter-rater reliability (IRR = 0.95), making this dataset a valid resource for further exploration and classification models.

In our six-step methodology, we applied a data augmentation technique to improve the prediction performance of the models. The challenges of collecting in-vehicle data in realistic settings have led researchers to work with small datasets, which usually present unbalanced class distribution. To deal with this issue, data-augmentation techniques have proven a cost-effective solution in generating similar datapoints. In our study, the classification results using the SMOTE technique are promising. As presented in Table 5, the evaluation metrics for the five models indicated that, using this technique, we could obtain a good model performance, with accuracies ranging from 0.88 to 0.92. To the best of our knowledge, this is the first work reporting this technique for in-vehicle data and driving-styles classification. This technique has been applied and proven effective in other research areas, such as medicine [73], image classification [74], and speech recognition [75], to name a few. We are aware that other data-augmentation techniques should also be considered in further studies.

In relation to the model design, the best way of designing prediction models is to tune the parameters and select the model that yields the best performance. This is done by performing a grid search on the parameters for each model, as explained in Section 3. In this way, we are exploring a large part of the search space (i.e., all possible models) by training and testing as many models as possible. Previous studies have also used grid search to tune model parameters, similar to our approach. For instance, Júnior et al. performed a grid search to tune SVM, RF, and ANN models parameters [14]. In this study, authors searched for the best SVM model by tuning the kernel function (linear, polynomial, RBF, sigmoid), C (2^−3^, 2^−1^, 2) and γ (2^−13^, 2^−11^, 2^−9^) parameters. Moreover, they looked for the best RF model by tuning the number of trees (*n*) from 200 to 100 and the number of layers (m) from 10 to 15. Finally, for the ANN model, they tuned the number of hidden layers from 40, 30, 20, and 10. The authors reported that the RF (n = 200, m = 15) model had the best performance (AUC = 0.985) among all other models generated using the grid search. Wang et al. also tuned the SVM model parameters through grid search [17]. C and γ values ranged from 2^−5^ to 2^10^ and 2^−21^ to 2^9^, respectively. The authors indicated that the SVM model with C = 2^7^ and γ = 2^−9^ had the best performance (accuracy = 0.65). As expected, the resulting models from previous works differ from our results (SVM RBF, C = 2^5^; γ = 2^−2^). An explanation of this can be related to the “no free lunch theorem” [76], which states that in order to compare several search algorithms, we need to use the same information. This, however, is not the case with our study, as the models from previous studies were implemented using different features (longitudinal acceleration and lateral acceleration [14]; average acceleration, braking, and turning [31]).

Regarding the model evaluation, it is worth noting that, as mentioned in Section 3, most of the previous studies considered only accuracy as a performance metric. The methodology followed in this work allowed us to expand our vision to select the best model by, first, choosing a model based on several performance metrics, and second, performing statistical tests for determining the predicting power of these models. We used accuracy, F1-score, AUC, and Kappa metrics to determine the model with the best performance and the Wilcoxon pair-test to determine if there were a significant difference in how the models predicted. The discussion of both model evaluations is detailed in the next section.

### 5.2. Performance and Statistical Evaluation

The experimental results (Table 5) revealed that the SVM model with RBF kernel (C = 2^5^; γ= 2^−2^) had a better performance (accuracy = 0.96) than the other four models. This result is consistent with previous studies reported by Xue et al. [18] and Wang et al. [17]. In Reference [18], authors indicated that the SVM model (accuracy = 0.917) outperformed RF, kNN, and ANN models using acceleration, relative distance, and relative velocity features for classifying normal and aggressive driving styles from 320 driving tests in a naturalistic setting. Similarly, Wang et al. [17] demonstrated that the SVM model with RBF kernel (accuracy = 0.772) was also better than a linear kernel (accuracy = 0.86) using speed and throttle opening features to classify normal and aggressive driving styles. It is worth noting that we cannot generalize the comparison of these models, because each model was built using different features, outputs, and conditions. However, we can see that our model had a higher accuracy than the models reported in previous studies [17,18]. Thus, we recommend that future researchers explore SVM models first if their task is to classify calm, normal, and aggressive driving styles.

Our results also indicated that the kNN model (k = 3) correctly classified 92% of the instances, lower than that of a previously reported similar model. For instance, the kNN model built by Vaitkus et al. [40] correctly classified 98% of 110 driving samples as normal or aggressive using longitudinal acceleration, vertical acceleration, and the covariance between longitudinal and lateral acceleration as features. Again, as mentioned above, we cannot compare both accuracy results because each model uses a different dataset. However, a possible explanation of the low performance between our model and the model presented in Reference [40] may be the differences in relation to the experimental conditions. The authors mentioned that the dataset used for the study was collected in a controlled setting (same season, same traffic conditions, and same route) and stated that this could make the classification task simpler by distinguishing normal from aggressive driving styles. By contrast, our study used data collected from two cities in a naturalistic setting, with similar traffic conditions, which makes the classification task more challenging.

Our RF model (n = 100; m = 4) yielded an accuracy of 0.92. This result, however, does not support previous research. Previous work has reported a higher model’s performance for a similar classification task. For instance, the RF model (n = 200, m = 10) presented by Júnior et al. [14] reported a higher accuracy (0.99) for classifying 69 aggressive and non-aggressive events using lateral and longitudinal acceleration in a real-world experiment. Further research should be undertaken to investigate whether adding lateral and longitudinal acceleration to our feature space improves the classification performance.

In terms of the fuzzy logic model performance, our results indicated that this model had an accuracy of 0.88. This result differs from previous works. For instance, the model presented by Castignani [13] reached an accuracy of 0.90 for classifying calm and aggressive driving styles, whereas the model presented by Dörr et al. [15] gave a performance of 0.68 for classifying comfortable, normal, and sporty driving styles. Due to the lack of information on how the models from previous studies were built, we cannot provide an explanation of this result. However, we are aware that the fuzzy-logic model’s performance depends on the domain expertise of humans to design fuzzy rules and membership functions, as well as to correctly interpret the fuzzy outputs for a particular classification task [77].

Finally, our ANN model (sigmoid; lr = 0.4; N = 4) gave an accuracy of 0.88. Contrasting this result with a previous ANN model (tanh, N = 2) presented by Cheng et al. [16], our model had slightly lower accuracy (0.892 for unseen data). The authors used acceleration, pedal angle, and speed features from data collected using a simulator to classify calm, normal, and aggressive driving styles on different types of road (e.g., flat, slope).

In addition to the average performance reported from the five metrics, we were also interested in analyzing the results of the inter-class classification, with an emphasis on the aggressiveness of drivers, as this can be extremely critical in realistic scenarios. Results from the intra-class metrics, as presented in Table 5, indicated that SVM also outperformed the evaluation metrics compared to the other four models (F1-score calm = 0.9524; normal = 0.9545; aggressive = 0.9714). Even though the testing dataset was unbalanced, SVM was able to predict 97% of aggressive drivers. Implications of this result may provide researchers with a model that can be applied to smart vehicles for early detection of unsafe driving styles.

Another point to highlight is the statistical test to determine whether there was a significant difference in the prediction power of these models. Results indicated that the ANN model (accuracy = 0.86) predictions differ from the other four models. Interestingly, this model had the worst performance of all models. Furthermore, the results from the statistical test also suggested that we have the freedom to choose any of the other four models for our classification purposes. However, we should consider more simplistic solutions, as pointed out by the Occam’s Razor principle [21]. This principle, in brief, states that the simplest solution should be considered from a pool of solutions, as this fades away unnecessary assumptions. Therefore, it is up to the researcher to define the characteristics of a simple yet robust model. For instance, in this work, we could argue that the number of parameters to tune or its interpretability characterizes the simplicity of a model. If the number of parameters defines the simplest model, the kNN should be selected among the four models. On the other hand, if the interpretability of the model is used to define the simplicity of the model, the fuzzy logic model could be chosen as the best solution, as it is built from a set of simple rules. The SVM, kNN, and RF models, can be considered more like a black box because of the difficulty of understanding how the internal logic works, mainly when working with higher dimensional spaces.

### 5.3. Limitations

Although the proposed methodology has successfully demonstrated a systematic evaluation of driving-styles models from data collected in naturalistic settings, it has certain limitations that should be noted. Although the experimental results revealed that the SVM model outperformed the other four models, we cannot generalize these results by arguing that this will be the best model for all driving-style classification problems. Instead, the aim of the methodology proposed was to guide researchers to make informed and well-grounded decisions on their model selection and evaluation. Further work should investigate the implications of the proposed methodology in other contexts. Another limitation is the small size of the dataset (50 drivers) and drivers’ characteristics. Drivers’ gender was not proportional (62% males, 38% females) and age groups were concentrated in two groups (24–34 years: 44%; 35–54 years: 38%). Drivers aged between 18 and 24 years old did not voluntarily participate. Due to these constraints in gender proportion and age groups, it was not possible to draw conclusions based on these characteristics. Although it is not recommended to develop classification models using small datasets due to the possible shortcomings of model generalization, this work overcomes that limitation with the application of a data augmentation technique for training purposes (n = 165). However, we can still not generalize our results, due to the small number of samples (n = 50) for testing the driving-styles models. Finally, another limitation of this work is that we did not consider external factors that could affect driving styles, such as weather conditions and time of day (day or night). Further work should explore the viability of improving the classification by including such variables.

## 6. Conclusions

In this work, we proposed a data-driven machine learning methodology for classifying driving styles. The methodology proposed in this work can be seen as a guideline to help researchers make informed decisions about which model is best for their classification problem and most relatable to driving-styles classification. The six-step methodology enables researchers to deal with data logged from diverse sensors, data validation from experts using a data-driven approach, unbalanced class distribution by generating an augmented dataset for training purposes, driving-style classification through different classifiers, and evaluation of the classifiers using ML metrics (e.g., accuracy, F1-score), and statistical tests analysis. This methodology was illustrated by classifying driving styles from data collected in two different cities in a naturalistic setting. We designed and implemented five computational models (fuzzy logic, SVM, ANN, kNN, and RF) in order to determine which had the best evaluation performance for classifying driving styles. On the one hand, the experimental results showed that SVM resulted in the best classifier to identify driving styles and, specifically, aggressive drivers. On the other hand, significance test results revealed that there was a significant difference in how ANN predicted driving styles. Both evaluations can help get an informed decision about which model is best for this task.

Further work should evaluate more computational models, such as Hidden Markov Models (HMM), Bayesian models, and Gaussian mixture models (GMM), which have been implemented in related research [35,78,79]. Other metrics can be considered to evaluate machine learning models, for example, robustness (if results are stable over time), scalability (if the model can handle significant volumes of data), or speed (time for training, test, and prediction). Of course, the evaluation metric chosen depends on the research aim. For instance, if researchers are motivated to explore the delivery of real-time feedback to drivers, the desirable performance should discern the robustness and speed of the models.

## Figures and Tables

**Table 1:** Thresholds defined for sudden acceleration and sudden brake events depending on the traffic-flow level.

Event Types	Traffic-Flow Levels		
	Low	Medium	High
EventAcc	a >= 2 m/s^2^	a >= 1.5 m/s^2^	a >= 1 m/s^2^
EventBrake	a <= 2 m/s^2^	a <= 1.5 m/s^2^	a <= 1 m/s^2^

**Table 2:** Rules defined from inputs variables.

Rules	Antecedents		Consequent	Weight
1	EventAcc AvgAcc	High High	aggressive	1
2	EventBrake AvgDec	High High	aggressive	1
3	AvgAcc	Low	calm	0.8
4	EventAcc EventBrake	High High	aggressive	1
5	AvgDec EventBrake	Low Low	calm	0.8
6	AvgAcc AvgDec	Normal Normal	normal	0.9
7	Traffic violations	High	aggressive	1
8	Traffic violations	Low	calm	0.8
9	Traffic violations	Normal	normal	0.9
10	EventAcc EventBrake	Normal Normal	normal	0.9

**Table 3:** Grid search values for Artificial Neural Networks (ANN).

Equations	Number of Neurons	Accuracy	Reference
$2\;x\;N_{i}$ + 1	3	0.84	[56]
$(N_{i}$$+N_{0}$)/2	4	0.86	[57]
$\sqrt{N_{i}\;x\;N_{0}\;}$	10	0.86	[58]
$2N_{i}$	12	0.84	[59]

**Table 4:** Grid search values for Support Vector Machines (SVM).

Parameters	Values
Kernel function	linear, polynomial, radial basis function (RBF), sigmoid
C	[2^−5^, 2^10^]
γ	[2^−5^, 2^10^]

**Table 5:** Evaluation metrics (accuracy, F1-score, Area Under the Curve (AUC), Kappa) for each model. ANN = Artificial Neural Networks; SVM = Support Vector Machines; RF = Random Forests; kNN = k-Nearest Neighbor.

Model	Accuracy	F1-Score	AUC	Kappa
Fuzzy Logic	0.8800	0.8840	0.9072	0.8106
ANN (sigmoid; lr = 0.4; N = 4)	0.8600	0.8663	0.9030	0.7807
SVM (RBF, C = 2^5^; γ = 2^−2^ )	0.9600	0.9595	0.9730	0.9375
RF (n = 100; m = 4)	0.9200	0.9253	0.9451	0.8750
kNN (k = 3)	0.9200	0.9253	0.9451	0.8750

**Table 6:** F1-score and AUC per driving style.

	Fuzzy Logic		ANN		SVM		KNN		RF	
Class	F1	AUC	F1	AUC	F1	AUC	F1	AUC	F1	AUC
Calm	0.9	0.9375	0.9091	0.9167	0.9524	0.9875	0.9524	0.9875	0.9524	0.9875
Normal	0.8696	0.8831	0.8511	0.8600	0.9545	0.9594	0.9091	0.9189	0.9091	0.9189
Aggressive	0.8823	0.9011	0.8387	0.8387	0.9714	0.9722	0.9143	0.9289	0.9143	0.9289

**Table 7:** Wilcoxon pair tests with corresponding *p*-value.

Classifier 1	Classifier 2	*p*-Value
SVM	ANN	0.025
SVM	Fuzzy	1
SVM	kNN	1
SVM	RF	1
ANN	Fuzzy	0.025
ANN	kNN	0.025
ANN	RF	0.025
Fuzzy	kNN	1
Fuzzy	RF	1
kNN	RF	1
